# Human mitochondrial glutathione transferases: Kinetic parameters and accommodation of a mitochondria-targeting group in substrates

**DOI:** 10.1016/j.bmc.2024.117712

**Published:** 2024-04-03

**Authors:** Patrick A. Cardwell, Carlo Del Moro, Michael P. Murphy, Adrian J. Lapthorn, Richard C. Hartley

**Affiliations:** a School of Chemistry, Joseph Black Building, University Avenue, University of Glasgow, Glasgow G12 8QQ, UK; b MRC Mitochondrial Biology Unit, University of Cambridge, Cambridge Biomedical Campus, Cambridge CB2 0XY, UK

**Keywords:** Glutathione-*S*-transferase, Glutathione, Mitochondria, Oxidative stress, Cancer

## Abstract

Glutathione-*S*-transferases are key to the cellular detoxification of xenobiotics and products of oxidative damage. GSTs catalyse the reaction of glutathione (GSH) with electrophiles to form stable thioether adducts. GSTK1-1 is the main GST isoform in the mitochondrial matrix, but the GSTA1-1 and GSTA4-4 isoforms are also thought to be in the mitochondria with their distribution altering in transformed cells, thus potentially providing a cancer specific target. A mitochondria-targeted version of the GST substrate 1-chloro-2,4-dinitrobenzene (CDNB), MitoCDNB, has been used to manipulate the mitochondrial GSH pool. To finesse this approach to target particular GST isoforms in the context of cancer, here we have determined the k_cat_/K_m_ for the human isoforms of GSTK1-1, GSTA1-1 and GSTA4-4 with respect to GSH and CDNB. We show how the rate of the GST-catalysed reaction between GSH and CDNB analogues can be modified by both the electron withdrawing substituents, and by the position of the mitochondria-targeting triphenylphosphonium on the chlorobenzene ring to tune the activity of mitochondria-targeted substrates. These findings can now be exploited to selectively disrupt the mitochondrial GSH pools of cancer cells expressing particular GST isoforms.

## 1 Introduction

GSTs are a major superfamily of enzymes which protect cells against toxicity caused by electrophiles such as xenobiotics and reactive metabolites generated by oxidative stress.^1–2^ The main function of GSTs is to catalyse conjugation of GSH, a small, ubiquitous thiol molecule, to reactive electrophiles, generating a less-reactive GS-conjugate for excretion from the cell. Some GST isoforms are often overexpressed in cancer cells causing drug resistance,^2–4^ for example to cisplatin,^5^ busulfan,^6^ and dichloroacetate,^7^ which reduces the efficacy of these drugs in anticancer therapy. These findings have encouraged the development of GST inhibitors as potential therapies.^3^ The overexpression of GSTs in cancer cells has also been utilised to metabolise GST-activated prodrugs to deliver high concentrations of an active drug to tumours, while reducing off-target effects.^2–4^ Current GST-activated anticancer prodrugs include; canfosfamide,^8^ which is in phase II and III trials to treat ovarian cancer;^9–10^ metformin derivatives,^11^ which exhibit potential to treat pancreatic and colon cancers; and doxorubicin derivatives, which overcome drug resistance to doxorubicin in solid tumours.^12^

Mitochondria are the home of the respiratory chain which is a major site of reactive oxygen species (ROS) production. Mitochondria have a number of pathways to protect against oxidative damage caused by these ROS. One of these is the mitochondrial GSH (mGSH) system.^13^ GSH is produced in the cytosol and transported into mitochondria,^14–15^ where it is used by GSTs and other antioxidant enzymes to support mitochondrial function.^2,13^ Thus, cells require a sustained mGSH pool to counter various types of damage, and disruption of this pool is thought to contribute to several pathologies, including cancer.^16–18^ Overproduction of mitochondrial ROS is a common property of many cancer cells, with elevated GSH levels also observed in a range of cancers, suggesting that elevated GSH may help survival of cancer cells by counteracting cell death pathways.^16,19–20^ Hence, one potential anti-cancer strategy is the selective disruption of the mGSH pool. Thus, here we have explored the ways in which the conjugation reactions of GST isoforms in the mitochondria can be tuned to enhance the efficacy and selectivity of this type of approach.

**Fig. 1:**
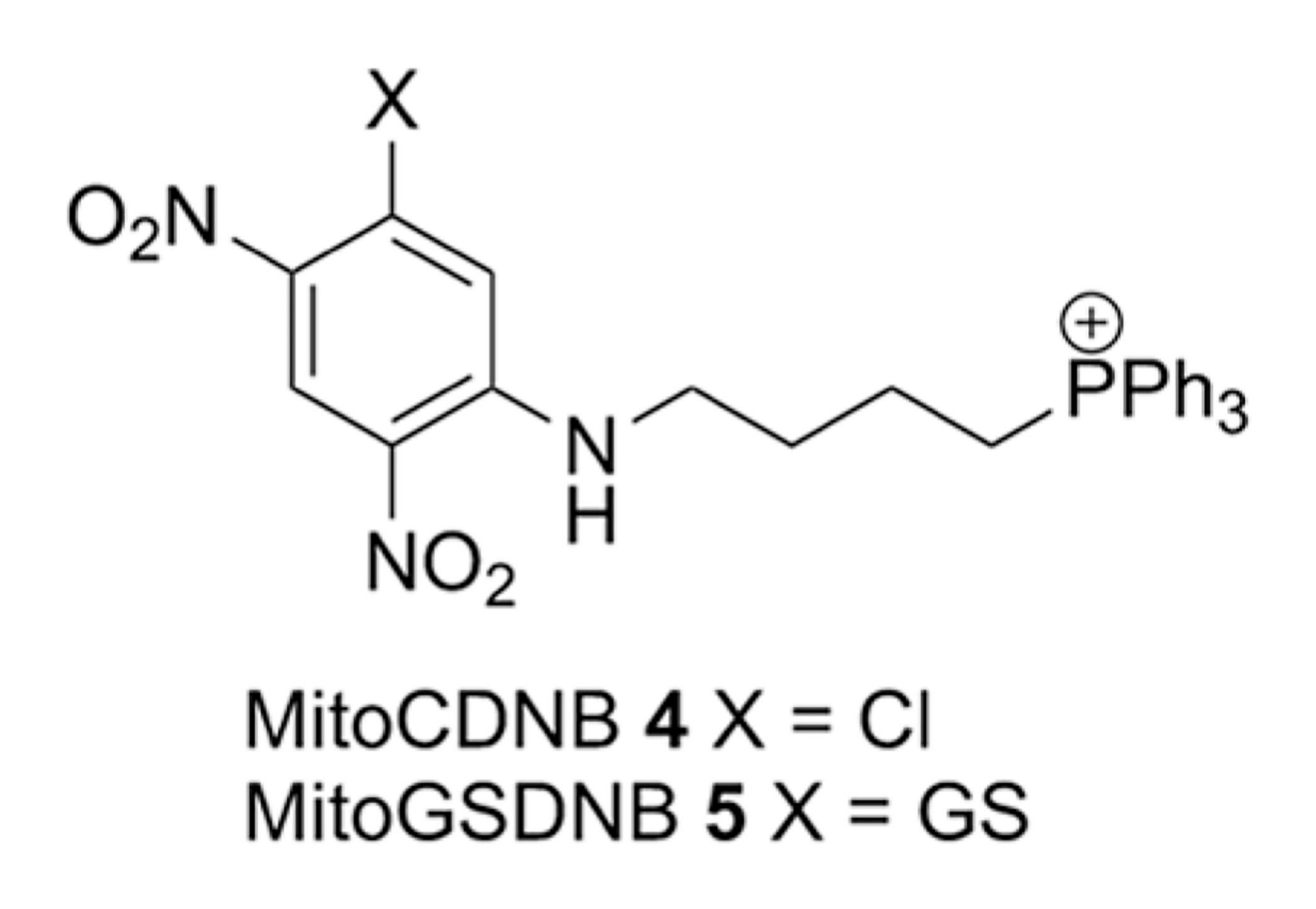
Structure of MitoCDNB **4** and its GSH adduct, MitoGSDNB **5**.

**Scheme 1:**
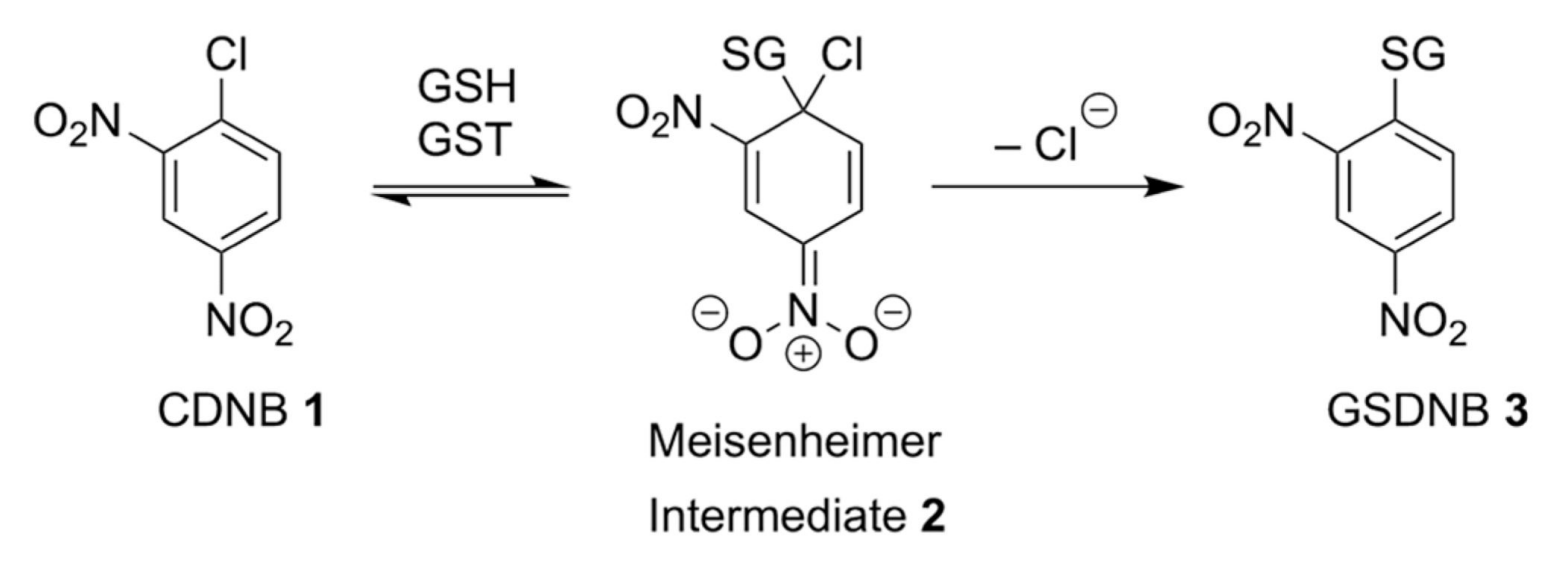
Mechanism for GST-catalysed nucleophilic aromatic substitution (S_N_Ar) of CDNB and GSH.

Many GSTs catalyse the conjugation of GSH to CDNB **1** (Scheme 1) by nucleophilic aromatic substitution (S_N_Ar) to form the GSDNB adduct **3**. Consequently, CDNB is routinely used as a substrate to test for the activity of GSTs. The mechanism is believed to follow a classical two-step addition–elimination S_N_Ar pathway, in which formation of the Meisenheimer intermediate **2** is the rate-determining step.^21^ Recently, we developed MitoCDNB **4** as a mitochondria-targeted analogue of CDNB **1** (Fig. 1).^22^ The CDNB moiety is linked to a triphenylphosphonium (TPP) cation, which enables the molecule to accumulate within the mitochondrial matrix, several-hundred fold, in response to the large negative-inside mitochondrial membrane potential.^22–24^ Mitochondrial GSTs catalyse the conjugation of mGSH to MitoCDNB **4** to produce MitoGSDNB **5**, which is rapidly exported from the mitochondria and cell. As a result MitoCDNB **4** selectively depletes the mGSH pool, as well as inactivating thioredoxin reductase 2.^22^ Since the mGSH pool is independent of the cytosolic GSH pool, it requires a long time to recover.^17^ This may mean that depletion of the mGSH pool has potential in cancer therapy, to selectively enhance oxidative damage and cell death of those cancer cells which already have high levels of mitochondrial ROS generation.

GSTs have two binding sites. A GSH-binding site (G-site), which is well-conserved among the different classes of enzymes, and a hydrophobic substrate-binding site (H-site), which can vary significantly.^25^ The main human mitochondrial GST is hGSTK1-1.^1,26^ GSTA1-1 and GSTA4-4 are also reported to be present in the mitochondrial matrix as well as the cytosol in humans and rodents.^27^ All these isoforms could potentially be exploited to selectively deplete mGSH by mGST-catalysed reaction with mitochondria-targeted electrophiles. Importantly, the mitochondrial localisation of GSTA1-1 and GSTA4-4 is reported to be increased under oxidative stress,^28–29^ such as that which occurs in cancer cells. Thus, differences in activity between these GST isoforms could potentially be exploited to maximise efficiency of mGSH depletion and selectively deplete mGSH according to the mGSTs present, or to selectively activate prodrugs, within cancer cells. Thus, we set out to characterise the key kinetic parameters that describe GST activity for GSH and CDNB **1**, for these three GST isoforms.

**Fig. 2:**
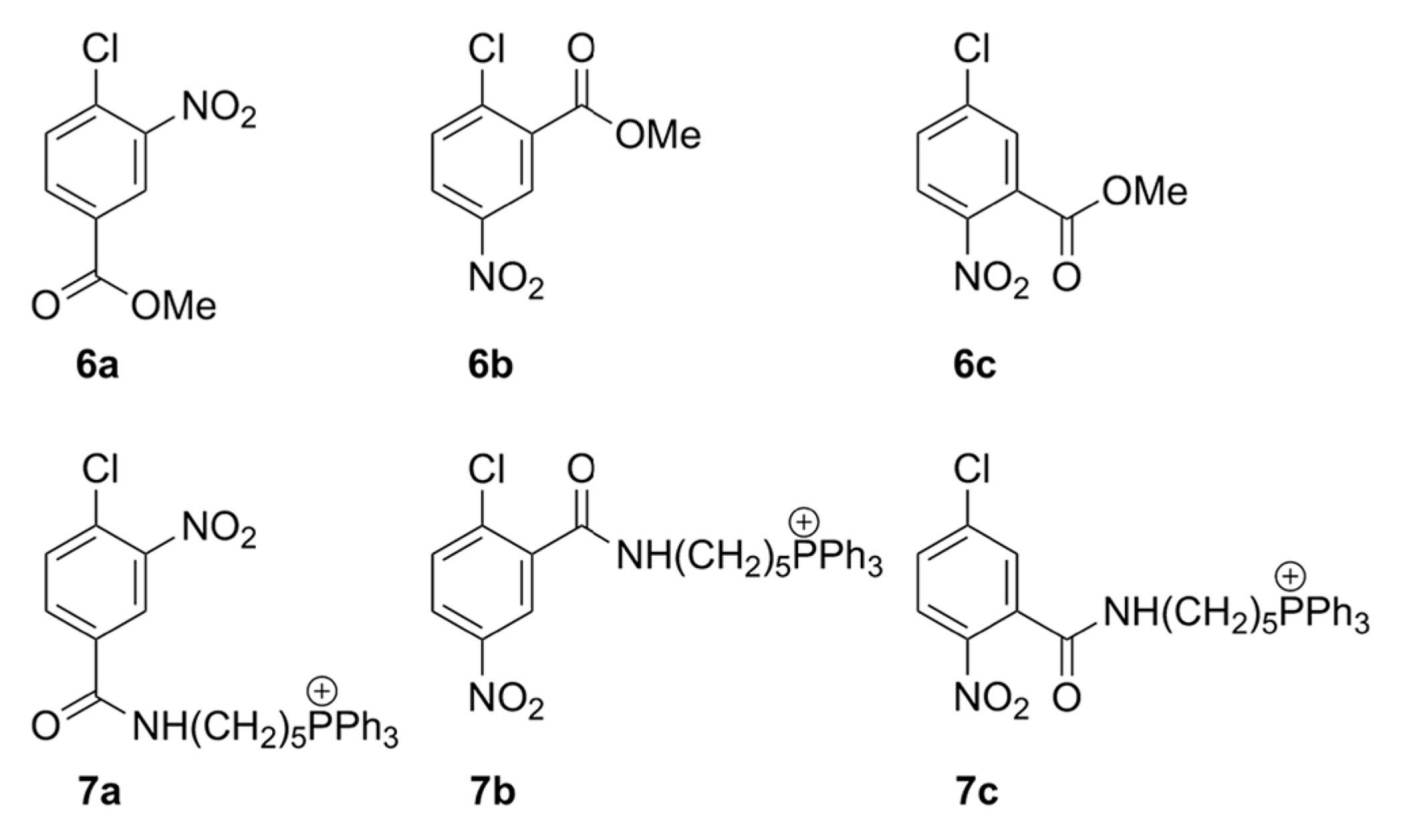
Structures of methyl esters **6** and TPP-pentyl amides **7** GST substrates investigated, grouped by substitution pattern, denoted by the letter.

We also wished to establish how well a mitochondria-targeting group could be accommodated at different sites in the CDNB structure and what factors affected the rate of GST-catalysed reaction of different CDNB analogues. While increasing substrate electrophilicity would likely deplete mGSH faster, it might also lead to unwanted side reactions with protein thiols. Rapid enzyme-catalysed reaction with low uncatalysed background rates would be ideal. Therefore, a series of electrophilic chloronitrobenzene derivatives **6** and **7** (Fig. 2) were prepared, based on the structures of CDNB **1** and MitoCDNB **4**, and the kinetics of their GST-catalysed GSH-conjugation were measured with each GST isoform, to determine which compounds were good substrates. The GSH adducts GSDNB **3** and MitoGSDNB **5** were also synthesised for these studies. Both the electronics and the position of the TPP-targeting group on the chloronitrobenzene substrate core were modified to determine how these might influence reactivity. S_N_Ar reactions are favourable when the electrophile contains electron-withdrawing groups (EWGs), such as nitro groups, at the *ortho*- and *para*-positions on the aromatic ring. In the chloronitrobenzene derivatives **6** and **7**, we replaced one nitro group for an electron-withdrawing ester group, or an electronwithdrawing amide group through which the TPP cation was attached. The position of substituents on the ring was varied to investigate the effects on the reactivity of the substrates for the GST-catalysed S_N_Ar reaction. The esters **6** would inform on intrinsic reactivity, while the TPP-amides **7** would be used to explore whether the enzyme active site had the space to accommodate the linker to the TPP targeting group.

## 2 Results and discussion

### 2.1 Synthesis of chloronitrobenzene derivatives and corresponding GSH-conjugates

**Scheme 2:**
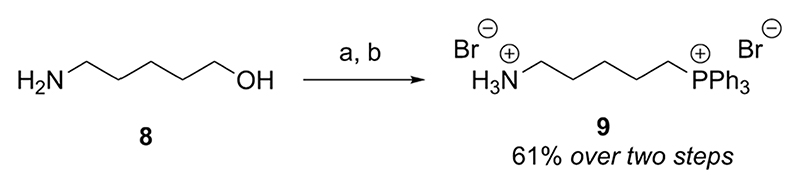
Reagents and conditions for the synthesis of TPP-amine linker as an ammonium salt **9**: (a) HBr (48 % w/v in water), reflux, 48 h; (b) Triphenylphosphine, MeCN, reflux, 48 h.

**Scheme 3:**
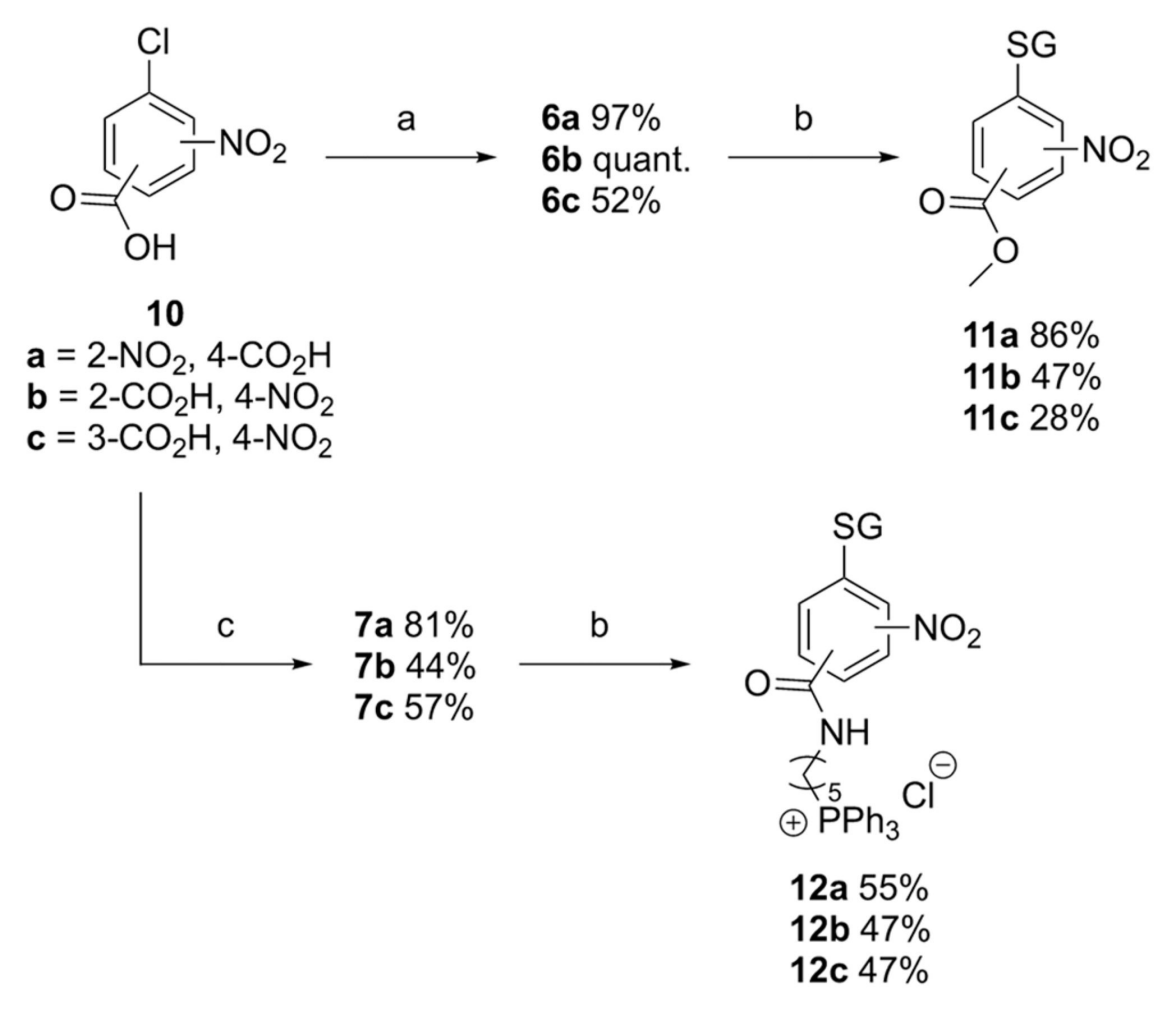
Reagents and conditions for the synthesis of chloronitrobenzene derivatives **6** and **7** and the corresponding GS-conjugates **11** and **12**: (a) H_2_SO_4_, MeOH, 60 °C, 20 h; (b) GSH, NaHCO_3_, EtOH, H_2_O, RT, 24 h; (c) i) DCC or EDCI, NHS, DCM, RT, 2 h; ii) **9**, NEt_3_, DCM, RT, 20 h. Substituents numbered relative to the chloro substituent.

MitoCDNB **4** and MitoGSDNB **5** were synthesised by the literature procedures.^22^ The TPP-amine used to synthesise amides **7 a-c** was prepared in two steps from the amino alcohol **8** and isolated as the ammonium salt **9** (Scheme 2). The chloronitrobenzene derivatives **6a-c** and **7a-c** were then synthesised from carboxylic acids **10a-c** by esterification and amide formation, respectively. The corresponding GS-conjugates **11a-c** and **12a-c** were prepared from these by reaction with the thiolate of GSH. GSDNB **3** was prepared from CDNB **1** in the same way. See (Scheme 3).

### 2.2 Kinetic parameters for CDNB and GSH at pH 6.5 and 8

Synthesised hGSTK1-1, hGSTA1-1 and hGSTA4-4 genes were purchased and cloned into an expression vector pNIC-28-*Bsa*I, which introduces 6 × histidine-tag and a tobacco etch virus (TEV) protease recognition sequence at the *N*-terminus of each protein to aid purification. The genes were expressed in *Escherichia coli* and the proteins purified via nickel affinity chromatography. Each GST enzyme was obtained in high yield with 28 mg of GSTK1-1, 59 mg of GSTA1-1, and 42 mg of GSTA4-4, recovered from 0.6 L cultures. SDS-PAGE analysis confirmed that the proteins were isolated in high purity. Next, the kinetic parameters for GST catalysed reaction between CDNB **1** and GSH were determined using UV absorbance assays, in which the concentration of one substrate was varied while the other was fixed at near saturating concentration. The formation of GSDNB **3** was monitored using the change in absorption at 340 nm (λ_max_, Δε_340nm_ = 9.02 mM^−1^cm^−1^). The data were then readily fitted to the Michaelis-Menten equation. Non-linear regression analysis gave values for the catalytic constant, *k*_*cat*_ and, the Michaelis-Menten constant, *K*_*M*_ for each substrate when its concentration was varied and the other was in excess (Table 1). It was possible to saturate all the GST isoforms with 10 mM GSH and so obtain accurate *k*_*cat*_ and *K*_*M*_ for CDNB **1**. However, only apparent *k*_*cat*_ and *K*_*M*_ could be obtained for GSH because CDNB **1** has poor water solubility and 1 mM was the highest concentration where we could be confident no precipitation occurred. Comparable concentrations of CDNB **1** have been used for the kinetic parameters reported in the literature.^30–31^ The standard protocol for GST-catalysed CDNB-GSH conjugation assays is carried out at pH 6.5 because the background rate is negligible,^32^ despite the fact that the majority of GST enzymes exhibit optimum activity above pH 7.0. We measured the kinetics of hGSTA1-1 and hGSTA4-4 at pH 6.5 to compare the kinetic parameters with those reported in the literature.^31,33–34^ Subsequently we measured the kinetics of all three enzymes at pH 8.0, the pH of the mitochondrial matrix. Kinetics for hGSTK1-1 were only measured at pH 8.0, emulating the methods of Robinson *et al*., whose studies revealed the enzyme’s optimum activity in the pH range 8.0–9.5.^31^

As expected, all three GST enzymes exhibited higher activity at pH 8.0 than at pH 6.5, but they showed markedly different activities for the two substrates. For both CDNB **1** and GSH, the order of enzyme activity was hGSTA1-1 > hGSTA4-4 > hGSTK1-1. The kinetic constants for hGSTA1-1 at pH 6.5 are comparable to those described by Zhao *et al*.^34^ The kinetic constants of hGSTA4-4 for CDNB **1** are broadly consistent with those of Hubatsch *et al*.,^35^ though the pH of their measurements is not stated.

The difference in k_cat_/K_M_ for CDNB **1** among the alpha class of GSTs, hGSTA1-1 and hGSTA4-4, is not surprising. Although these have 76 % sequence similarity and 54 % sequence identity to each other they have previously been shown to have very different substrate specificity.^36–40^ hGSTA1-1 exhibits significant catalytic activity towards substrates of diverse structural compositions, while hGSTA4-4 is distinguished by its specificity for alkenals, particularly 4-hydroxynonenal (HNE) a toxic end-product of oxidative stress.^36–40^ This difference in substrate specificity has been explored by various groups by site directed mutagenesis and structural studies. The H-site of hGSTA1-1 and hGSTA4-4 is formed from three regions of the protein. These are the β1-α1 loop (residues 9–16), the C-terminal part of the α4 helix (residues 104–111), and the α9 helix at the C terminus of the protein (residues 210–220).^41^ In particular, the C-terminal α9-helix has been revealed by numerous studies to play a crucial role in governing the specificities of these enzymes.^36–39^ GSTA1-1 has a highly heterogeneous C-terminal conformation which allows the H-site to tolerate a variety of substrates.^41^ In addition probes of molecular flexibility and molecular dynamics simulations to compare the two enzymes have shown increased global solvent exchange properties and apparent flexibility in the protein core of hGSTA1-1 and at residues far from the active site.^36^ A mutant, hGSTA1–1 GIMFhelix, has been created by introducing the hGSTA4-4 residues 12, 107, 108, and 111 and the C-terminal peptide (residues 208–222) into the structure of hGSTA1-1. This conferred significant hGSTA4-4 substrate specificity with a 300-fold increase in catalytic efficiency with HNE and a > 10 times decreased activity with CDNB.^38^

The kappa class enzyme hGSTK1-1 is part of a distinct family of thioredoxin fold proteins, which share more in common with 2-hydroxychromene-2-carboxylic acid (HCCA) isomerase and disulfide-bond-forming DsbA oxidoreductase found in bacteria than with other cytosolic GSTs.^31,33,42^ The G site of hGSTK1-1 utilises many of the same key amino acids for binding to GSH as cytosolic GSTs, with some minor differences, but the H-site is strikingly different.^33^ Structural studies on hGSTK1-1 show that in the apo enzyme both the G and H sites are accessible to the solvent allowing random binding of substrate and GSH. However, on the binding of GSH analogues loops α2-α3 and α3-α4 of the H-site become ordered closing the active site although these regions are still mobile.^33^ Like hGSTA1-1 the H site seems to adopt different conformations in the presence of different GSH conjugates, however this does not confer broad substrate promiscuity in the case hGSTK1-1.

For the hGSTK1-1 enzyme, we observed hyperbolic Michaelis-Menten character for hGSTK1-1 for both CDNB **1** and GSH, which is in agreement with Wang *et al*.,^33^ but in contrast to Robinson *et al*.,^31^ who detected hyperbolic Michaelis-Menten character only for GSH and sigmoidal character for CDNB **1**. Our kinetic constants are similar to those reported by Wang *et al*.,^33^ and indicate that hGSTK1-1 has low affinity for CDNB **1** and moderate affinity for GSH, which may suggest that hGSTK1-1 has better GSH-conjugating activity for other substrates. By comparison, Robinson *et al*., report low affinities for both CDNB **1** and GSH.^31^ Interestingly, murine GSTK1-1 and rat GSTK1-1 show a significantly higher specific activity for CDNB **1** than the human isoform and many other GSTs of the same species.^43^ Comparison of protein sequences demonstrate that murine GSTK1-1 and rat GSTK1-1 share 86 % identity,^43^ but only a 71 % and 69 % identity, respectively, with hGSTK1-1.^26^ The G site of hGSTK1-1 utilises many of the same key amino acids for binding to GSH as cytosolic GSTs, with some minor differences, but the H-site is strikingly different.^33^.

The results of our Michaelis-Menten kinetic studies for the common substrates CDNB **1** and GSH provide a direct comparison between the activity of the three different hGST enzymes, most likely involved in mitochondrial antioxidant defence. Our kinetic parameters for hGSTA1-1 are consistent with those found in the literature and confirm that GSTA1-1 is a highly active enzyme for electrophilic compounds that undergo S_N_Ar reactions. Our kinetic characterisation of hGSTA4-4 confirms that this enzyme is significantly less active for CDNB consistent with this enzyme’s high activity for Michael-addition-type electrophiles such as HNE. hGSTK1-1 is a structurally distinct GST with its own unique substrate specificity and our kinetics largely agree with those reported in the literature. We have extended the kinetic characterisation to pH 8.0 the pH of the mitochondrial matrix. These kinetics serve as a platform to investigate a range of CDNB related compounds to explore the scope for specific GSH depletion within the mitochondrial matrix.

### 2.3 Comparison of GST catalysis of different substrates at pH 8

Next we wished to assess how different substituents affected the catalysis of conjugation to GSH by the three hGSTs. We decided to measure the initial rates of reaction at pH 8.0 of the substrates **1, 4, 6a-c** and **7a-c** at variable concentrations with 10 mM GSH at 37 °C in the presence of the different enzymes. As before, GSH would be used in great excess (>15 fold the apparent *K*_*M*_ for GSH for each enzyme) so that pseudo first order reaction can be observed with respect to the substrate **1, 4, 6a-c** and **7a-c**. In each case, reactions would be monitored using the increase of absorption at the λ_max_ wavelength of the GS adduct **3, 5, 11a-c** and **12a-c**, respectively (see experimental). These did not overlap with absorption by GSH or the corresponding chloronitrobenzene substrate **1, 4, 6a-c** and **7a-c**.

First the rate of the uncatalysed reactions were determined under the conditions above. Table 2 presents these data as the background rate and the rate relative to CDNB **1**. An apparent second order rate constant is inferred on the assumption that the reaction is second order for all substrates in line with the well-established mechanism of this type of S_N_Ar reaction and the observed *pseudo* first order kinetics for the reaction with CDNB **1** at different concentrations. Only three compounds **1, 4**, and **6a** showed detectable background reaction. The relative reactivity of compounds with a nitro group *ortho* to the chloro substituent is *para*-nitro **1** > *para*-carboxylic ester **6a** > *para*-amide **7a**. The results contrast with those of Miller *et al*, who found very similar amides to amide **7a** were more susceptible to S_N_Ar reaction than ester **6a** when reacted with sodium methoxide in methanol.^44^ Potentially the difference is a result of using a soft nucleophile rather than the hard methoxide nucleophile. The trend in our results is consistent with the Hammett parameters for conjugating electron-withdrawing *para* substituents stabilising a negative charge: $\sigma_{\text{p}}^{-}=+1.27$ for a nitro group, $\sigma_{\text{p}}^{-}=+0.75$ for CO_2_Me and $\sigma_{\text{p}}^{-}=+0.61$ and + 0.70 for CONH_2_ and CONMe_2_, respectively.^45^ The result is expected because both the *ortho* and *para* electron-withdrawing groups stabilise the Meisenheimer intermediate by direct conjugation and hence also stabilise the negative charge developing in the transition state of the rate determining step that leads to this intermediate. Electron donation from the amino group in MitoCDNB **4** weakens electron-withdrawal to the nitro groups, so that MitoCDNB **4** is less reactive than CDNB **1**, though it is still more reactive than ester **6a**.

When the nitro and ester groups of compound **6a** are switched, the resulting compound **6b** is unreactive. The nitro group has a greater effect when *ortho* because it supplements resonance stabilisation with strong electron-withdrawal by induction. This induction is greater than for the ester and amide groups as evident from the Hammett parameters for *meta* substituents where resonance is not possible: σ_m_ = +0.71 for a nitro group, σ_m_ = +0.37 for CO_2_Me and σ_m_ = +0.35 for CONHMe.[45] The low inductive effect of the carbonyl groups explains why compounds **6c** and **7c** are also unreactive. Although, GSH is mostly in its thiol form at pH 8, reaction will occur via the thiolate because it is by far the most nucleophilic species present and about one tenth of the GSH is in this deprotonated form (the thiol pKa = 8.94 or 9.27 depending on whether the amino group is protonated or not).^46^ Solvation of the thiolate by coordination to the NH amide or activation of the thiol by coordination to the carbonyl or nitro group oxygen atom are not important to the rate of reaction in water, which solvates both positive and negative charges well.^47^ Next the reactivity of the different substrates **1, 4, 6a-c** and **7a-c** was studied in GST-catalysed reactions (Table 3). The formation of the GS adducts **3, 5, 11a-c** and **12a-c** was monitored in the same way as before. The background rates were subtracted from observed reaction rates to obtain the enzyme-catalysed rates. The kinetic parameters were then calculated.

Several conclusions can be drawn from the *k*_*cat*_/*K*_*M*_ data for chloronitrobenzenes **1, 4, 6a-c** and **7a-c** with the different GST isoforms. Firstly, hGSTA1-1 is the most active enzyme for all the substrates, highlighting its promiscuity for electrophilic compounds that undergo S_N_Ar reactions. The activities of hGSTA4-4 and hGSTK1-1 are significantly lower and more comparable with one another. These results complement the findings from the Michaelis-Menten kinetics on CDNB **1**.

In line with the uncatalysed reaction, substrates **1, 4, 6a** and **7a** that have a nitro group *ortho* to the chloro substituent are more reactive than the chloronitrobenzene derivatives **6b,c** and **7b,c** where the nitro group is *para*. As discussed above, this reflects the combination of strong inductive and mesomeric electron withdrawal by the *ortho* nitro group stabilising the negative charge in the Meisenheimer complex. Again hydrogen bonding between an *ortho* nitro group and the nucleophile^47^ is unlikely to be a component in this high rate of reaction because this would have to compete with the specific interactions between the SH of the GSH and the GST which lowers its pKa to 6 or 7.^21^

MitoCDNB **4** is a particularly good substrate for hGSTA1-1 and hGSTA4-4 (Table 3). The hGSTA1-1 catalysed reaction of CDNB **1** is 3.5 times faster than the catalysed reaction of MitoCDNB **4**, but this appears to be simply the result of CDNB **1**′s greater electrophilicity: the 3-fold higher *k*_*cat*_ for CDNB **1** (Table 4) mirrors the 3.4-fold higher background rate (Table 2). There is little difference in the *K*_*M*_ of the two substrates (Table 5). Interestingly, MitoCDNB **4** is a marginally better substrate than CDNB **1** for hGSTA4-4. The *k*_*cat*_’s are surprisingly similar for the two substrates **1** and **4**. It may be that the alkyl chain prevents MitoCDNB **4** binding in unproductive conformations accessible to the smaller CDNB **1**, negating the effect of the latter’s greater electrophilicity. MitoCDNB **4** is 23 times more reactive than ester **6a** in the hGSTA1-1 catalysed reaction, and 221 times faster as a substrate for hGSTA4-4 (Table 3). The *k*_*cat*_’s for MitoCDNB **4** are 3- and 9-times those for ester **6a** for hGSTA1-1 and hGSTA4-4, respectively (Table 4). The difference corresponds well to the 5-fold greater reactivity of MitoCDNB **4** in the background reaction (Table 2), and so reflects the relative electrophilicity of the two substrates **4** and **6a**. The main reason that MitoCDNB **4** is a better substrate than the ester **6a** is that it binds more strongly to the two enzymes, having a 14-fold lower *K*_*M*_ for hGSTA1-1 and a 25-fold lower *K*_*M*_ for hGSTA4-4. Stronger binding may simply be the hydrophobic effect of the alkyl chain, but it is clear that the alkyl chain is well accommodated at C(5). In contrast, *k*_*cat*_/*K*_*M*_ for the hGSTK1-1-catalysed reaction of MitoCDNB **4** is 28 times slower than CDNB **1** and similar to the ester **6a**, indicating that the C(5) chain is not well accommodated by hGSTK1-1. This difference in catalysis combined with the fact that chloronitrobenzenes are generally worse substrates for hGSTK1-1 may mean that MitoCDNB **4** will selectively deplete mGSH from cells where hGSTA1-1 and hGSTA4-4 are imported into the mitochondria in response to oxidative stress.

Attaching the TPP-alkyl chain through a C(3) amide gives a slightly better substrate than the ester **6a** counter to expectations from the Hammett parameters and the observed uncatalysed reaction. However, accommodation of the chain at this site does not compensate for the less favourable weak electron withdrawal of a *para* amide compared to the *para* nitro group in CDNB **1**. Attaching the alkyl chain at the *ortho* position appears to confer no advantages. The amide **7b** is less reactive than the ester **6b** as expected both from the Hammett parameters and from the rates of S_N_Ar reactions on ester **6b** and amides similar to amide **7b** with sodium methoxide in methanol reported by Miller et al.^44^

The particular interactions which make MitoCDNB **4** such a good substrate of hGSTA1-1 or hGSTA4-4 are not yet clear. However, crystallographic studies on mu and pi class GSTs report that H-bonding interactions with key tyrosine residues stabilise the Meisenheimer complex of CDNB **1**.^48–50^ A comprehensive study on hGSTA1-1 indicates that Arg-15, which is located at the interface of the G and H sites of many alpha class GSTs, has a crucial role in reducing the thiol’s pK_a_ and further stabilising the Meisenheimer complex (in addition to stabilisation from the interactions with tyrosine residues).^51^ Unfortunately, there are no aromatic substrate-bound hGSTA4-4 or hGSTA1-1 crystal structures. Docking studies on hGSTK1-1 in complex with GSDNB, suggest that hydrophobic interactions between the CDNB moiety and several hydrophobic residues in the H-site are important in substrate binding.^33^.

## 3 Conclusion

Our kinetic measurements demonstrate that the three mitochondrial GST isoforms can bind to and catalyse GSH-conjugation of several different electrophilic substrates. The reactivity of a substrate benefits from a highly electron-deficient aromatic system, as seen for CDNB **1** and MitoCDNB **4**. When the TPP alkyl chain is attached *meta* or *para* in MitoCDNB **4** and amide **7a**, respectively, it increases the GSH conjugation rate of hGSTA1-1 and GSTA4-4, but it confers no advantage when placed *ortho*. MitoCDNB **4** is a particularly good substrate for both hGSTA1-1 and hGSTA4-4, but GSH conjugation by hGSTK1-1 is three orders of magnitude less efficient. Hence, MitoCDNB **4** is an interesting lead compound for the cell selective depletion of mGSH through GST activity in cancer cells in which the GST isoforms expressed differs from non-transformed cells.

## 4 Experimental

### 4.1 Chemical Synthesis

#### 4.1.1 General information

All reagents were obtained from commercial suppliers. All reactions were performed under an inert argon atmosphere in oven- or flame-dried flasks. Anhydrous solvents were purified using a PurSolv 500 MD solvent purification system and added via syringe. ^1^H, ^13^C and ^31^P NMR spectra were obtained on a Bruker AVIII or DPX spectrometer operating at 400, 101, and 162 MHz, respectively. All coupling constants were measured in Hertz. Deuterated solvents contained trimethylsilane (TMS) as a reference compound. DEPT was used to assign the signals in ^13^C NMR spectra as C, CH, CH_2_ and CH_3_. Mass spectra (MS) were recorded on a Bruker MicroTOFq spectrometer for low and high resolution ESI^+^. A Shimadzu FTIR-8400S spectrometer was used to obtain infrared (IR) spectra. Melting points were obtained using a Gallenkamp melting point apparatus. UV–visible spectra and absorbance kinetics were recorded on a Jasco V550 UV/Vis spectrometer. Purification by flash chromatography used Biotage® IsoleraTM One Flash Chromatography system with Biotage® SNAP Ultra, Biotage® KIP or Agela silica gel cartridges.

#### 4.1.2 General procedure for preparation of GS-conjugates

MitoGSDNB **5** previously prepared by S. T. Caldwell for Booty *et al*.,^22^ was used in this study. Other CDNB adducts **3, 11** and **12** were prepared by adapting the procedure from Booty *et al*.^22^ Glutathione (1.50 eq.) was added to a solution of the chloronitrobenzene derivative (1.00 eq.) and NaHCO_3_ (3.00 eq.) in EtOH:H_2_O (1:1 v/v, 0.1 M in CDNB and esters, 0.05 M in amides). The reaction mixture was stirred at room temperature for 24 h or until the reaction was deemed complete. The reaction mixture was concentrated *in vacuo* and purified using RP-flash column chromatography.

#### 4.1.3 S-(2,4-Dinitrophenyl)glutathione (GSDNB) 3

The reaction was performed as described in the general procedure using CDNB **1** (110 mg, 0.494 mmol). The reaction mixture was stirred at room temperature for 24 h. Purification by RP-flash column chromatography [water:acetonitrile 0–100 %] afforded GSDNB **3** as an orange solid (227 mg, 97 %). υ_max_ (ATR): 1736 (C–O), 1726 (C–O), 1588 (ArC = ArC), 1511 (NO_2_) cm^−1^. ^1^H NMR (400 MHz, D_2_O): δ_H_ 9.12 – 9.06 (1H, m, H-3), 8.54 – 8.46 (1H, m, H-5), 7.95 – 7.87 (1H, m, H-6), 4.87 – 4.75 (1H, m, SCH_2_*CH*), 3.87 – 3.62 (4H, m, *CH*_*2*_CO_2_H, NH_2_*CH*, and S*CH*_*A*_), 3.47 (1H, dd, *J* = 14.2, 9.3 Hz, S*CH*_*B*_), 2.47 (2H, t, *J* = 7.8 Hz, NHCO*CH*_*2*_), 2.08 (2H, q, *J* = 7.4 Hz, NH_2_CH*CH*_*2*_). ^13^C NMR (101 MHz, D_2_O): δ_C_ 176.14 (C), 174.99 (C), 174.56 (C), 170.96 (C), 145.37 (C), 144.44 (C), 144.28 (C), 128.14 (CH), 127.71 (CH), 121.89 (CH), 54.18 (CH), 51.55 (CH), 43.36 (CH_2_), 33.51 (CH_2_), 31.45 (CH_2_), 26.53 (CH_2_). HRMS (ESI^+^): C_16_H_20_N_5_O_10_S requires 474.0925 found 474.0931 (MH^+^). Assignment of ^1^H and ^13^C signals was supported by analysis of COSY, HSQC and DEPT experiments. The ^1^H and ^13^C NMR data was in broad agreement with that previously reported in DMSO.^52^.

#### 4.1.4 {4-[(5′-chloro-2′,4′-dinitrophenyl)amino]butyl} triphenylphosphonium chloride (MitoCDNB) 4

Following the procedure from Booty *et al*.,^22^ anhydrous *N,N*-diisopropylethylamine (842 μL, 4.84 mmol, 2.00 eq.) was added dropwise to 1,5-dichloro-2,4-dinitrobenzene (631 mg, 2.66 mmol, 1.10 eq.) and (4-aminobutyl)triphenylphosphonium bromide (1.00 g, 2.42 mmol, 1.00 eq.) in acetonitrile (5.0 mL), and the reaction mixture was stirred for 24 h at room temperature under argon. The solvent was concentrated *in vacuo* and the solid redissolved in dichloromethane (40 mL). The organic layer was washed with HCl (30 mL, 1 N) and the aqueous layer was extracted with dichloromethane (3 × 25 mL). The organic layers were combined, dried with MgSO_4_ and the solvent concentrated *in vacuo*. The crude material was purified via column chromatography [SiO_2_, dichloromethane:methanol 0–20 %] to afford MitoCDNB as an orange foam (1.26 g, 83 %). υ_max_ (ATR): 3357 (N–H), 1609 (ArC = ArC), 1568 (NO_2_), 1538 (NO_2_) cm^−1^. ^1^H NMR (400 MHz, CDCl_3_): δ_H_ 8.93 (1H, s, H-3′), 8.42 (1H, t, *J* = 5.4 Hz, NH), 7.91 – 7.83 (6H, m, Ph), 7.82 – 7.74 (3H, m, Ph), 7.73 – 7.65 (6H, m, Ph), 7.16 (1H, s, H-6′), 4.01 (2H, m, CH_2_-1), 3.72 (2H, q, *J* = 6.5 Hz, CH_2_-4), 2.26 – 2.04 (2H, qn, *J* = 7.0 Hz, CH_2_-3), 1.94 – 1.65 (2H, m, CH_2_-2). ^13^C NMR (101 MHz, CDCl_3_): δ_C_ 146.90 (C), 136.11 (C), 135.27 (d, *J* = 3.1 Hz, 3 x CH), 134.56 (C), 133.83 (d, *J* = 10.0 Hz, 6 x CH), 130.67 (d, *J* = 12.6 Hz, 6 x CH), 128.97 (C), 126.81 (C), 118.57, 117.35 (d, *J* = 73.9 Hz, 3 x C), 42.73 (CH_2_), 28.85 (d, *J* = 17.0 Hz, CH_2_), 22.58 (d, *J* = 50.9 Hz, CH_2_), 19.86 (d, *J* = 4.0 Hz, CH_2_). ^31^P NMR (162 MHz, CDCl_3_): δ_P_ 24.68 (1P, s). HRMS (ESI^+^): ${\text{C}}_{28}{\text{H}}_{26}^{35}{\text{ClN}}_{3}{\text{O}}_{4}\text{P}$ requires 534.1344 found 534.1345 (M^+^). The ^1^H and ^13^C NMR data was in agreement with that previously reported.^22^.

#### 4.1.5 Methyl 4-Chloro-3-nitrobenzoate 6a

Following the procedure from Haydon *et al*.,^53^ sulfuric acid (400 μL) was added to 4-chloro-3-nitrobenzoic acid **10a** (2.50 g, 12.4 mmol) in methanol (15 mL) and the reaction mixture was stirred for 20 h at 60°C. The solvent was concentrated *in vacuo* and the crude material redissolved in ethyl acetate (30 mL). The organic layer was washed with water (50 mL) and the aqueous layer was extracted with ethyl acetate (3 × 30 mL). The organic layers were combined, dried with MgSO_4_ and the solvent concentrated *in vacuo* to afford the ester **6a** as a white solid (2.60 g, 97 %). Mp 77–78 °C. υ_max_ (ATR): 1714 (C=O), 1604 (ArC = ArC), 1537 (NO_2_) cm^–1^. ^1^H NMR (400 MHz, CDCl_3_): δ_H_ 8.52 (1H, d, *J* = 2.0 Hz, H-2), 8.17 (1H, dd, *J* = 8.4, 2.0 Hz, H-6), 7.65 (1H, d, *J* = 8.4 Hz, H-5), 3.97 (3H, s, CH_3_). ^13^C NMR (101 MHz, CDCl_3_): δ_C_ 164.37 (C), 148.06 (C), 133.73 (CH), 132.35 (CH), 131.86 (C), 130.20 (C), 126.77 (CH), 53.09 (CH_3_). Mass spectrometry by ESI^+^ failed to find MH^+^. The ^1^H and ^13^C NMR data was in agreement with that previously reported.^53^.

#### 4.1.6 Methyl 2-Chloro-5-nitrobenzoate 6b

Adapting the procedure from Haydon *et al*.,^53^ a solution of 2-chloro-5-nitrobenzoic acid **10b** (500 mg, 2.48 mmol) and sulfuric acid (0.20 mL) in methanol (7.5 mL) was stirred for 20 h at 60°C. The solvent was concentrated *in vacuo* and the crude material redissolved in dichloromethane (20 mL). The organic layer was washed with HCl (20 mL, 1 N) and the aqueous layer was extracted with dichloromethane (2 × 15 mL). The organic layers were combined and dried with MgSO_4_, and the solvent concentrated *in vacuo* to afford the ester **6b** as a white solid (541 mg, quant.). Mp 66–67°C. υ_max_ (ATR): 1729 (C=O), 1608 (ArC = ArC), 1573 (NO_2_), 1522 (NO_2_) cm^–1^. ^1^H NMR (400 MHz, CDCl_3_): δ_H_ 8.68 (1H, d, *J* = 2.7 Hz, H-6), 8.25 (1H, dd, *J* = 8.8, 2.7 Hz, H-4), 7.64 (1H, d, *J* = 8.8 Hz, H-3), 3.98 (3H, s, CH_3_). ^13^C NMR (101 MHz, CDCl_3_): δ_C_ 164.02 (C), 146.21 (C), 140.83 (C), 132.45 (CH), 131.08 (C), 126.92 (CH), 126.71 (CH), 53.17 (CH_3_). Mass spectrometry by ESI^+^ failed to find MH^+^. The ^1^H and ^13^C NMR data was in agreement with that previously reported. ^54^

#### 4.1.7 Methyl 5-Chloro-2-nitrobenzoate 6c

Adapting the procedure from Haydon *et al*.,^53^ a solution of 5-chloro-2-nitrobenzoic acid **10c** (1.00 g, 6.96 mmol) and sulfuric acid (0.40 mL) in methanol (15 mL) was stirred for 20 h at 60°C. The solvent was concentrated *in vacuo* and the crude material redissolved in dichloromethane (20 mL). The organic layer was washed with HCl (20 mL, 1 N) and the aqueous layer was extracted with dichloromethane (2 × 15 mL). The organic layers were combined and dried with MgSO_4_, and the solvent concentrated *in vacuo*. The crude material was purified by column chromatography [SiO_2_, hexane:dichloromethane 20–80 %] to afford ester **6c** as a white solid (774 mg, 52 %). Mp 48–49°C. υ_max_ (ATR): 1733 (C=O), 1567 (NO_2_), 1523 (NO_2_) cm^−1^. ^1^H NMR (400 MHz, CDCl_3_): δ_H_ 7.90 (1H, d, *J* = 8.7 Hz, H-3), 7.68 (1H, d, *J* = 2.3 Hz, H-6), 7.58 (1H, dd, *J* = 8.7, 2.3 Hz, H-4), 3.93 (3H, s, CH_3_). ^13^C NMR (101 MHz, CDCl_3_): δ_C_ 164.85 (C), 146.25 (C), 139.80 (C), 131.71 (CH), 129.93 (CH), 129.49 (C), 125.59 (CH), 53.69 (CH_3_). Mass spectrometry by ESI^+^ failed to find MH^+^. The ^1^H and ^13^C NMR data was in agreement with that previously reported.^55^.

#### 4.1.8 {5-[(4′-Chloro-3′-nitrophenyl)formamido]pentyl} triphenylphosphonium chloride 7a

*N, N*-dicyclohexylcarbodiimide (506 mg, 2.45 mmol, 1.25 eq.) was added to a stirring solution of 4-chloro-3-nitrobenzoic acid **10a** (396 mg, 1.96 mmol, 1.00 eq.) and *N*-hydroxysuccinimide (249 mg, 2.16 mmol, 1.10 eq.) in dichloromethane (8 mL) at 0°C. The reaction mixture was allowed to warm to room temperature and stirred for 2 h. A solution of (5-aminopentyl)triphenylphosphonium bromide hydrobromide **9** (1.00 g, 1.96 mmol, 1.00 eq.) and triethylamine (547 μL, 3.92 mmol, 2.00 eq.) in dichloromethane (2.0 mL) was added and the reaction mixture was stirred for 19 h at room temperature. The reaction mixture was filtered, and the filtrate was washed with an aqueous solution of HCl (20 mL, 1 N). The aqueous layer was extracted with dichloromethane (3 × 10 mL) and the organic layers were combined, dried with MgSO_4_, and the solvent concentrated *in vacuo*. The crude material was purified by column chromatography [SiO_2_, dichloromethane: methanol 0–20 %] to afford amide **7a** as a pale-yellow foam (906 mg, 81 %). υ_max_ (ATR): 3217 (N–H), 1651 (C=O), 1531 (NO_2_) cm^−1^. ^1^H NMR (400 MHz, CDCl_3_): δ_H_ 9.34 (1H, t, *J* = 5.6 Hz, NH), 8.78 (1H, dd, *J* = 8.4, 2.1 Hz, H-6′), 8.62 (1H, d, *J* = 2.1 Hz, H-2′), 7.86 – 7.65 (15H, m, Ph), 7.59 (1H, d, *J* = 8.4 Hz, H-5′), 3.67 – 3.55 (2H, m, CH_2_-1), 3.49 (2H, q, *J* = 5.8 Hz, CH_2_-5), 1.88 – 1.70 (6H, m, CH_2_-2, CH_2_-3 and CH_2_-4). ^13^C NMR (101 MHz, CDCl_3_): δ_C_ 164.32 (C), 147.72 (C), 135.18 (d, *J* = 3.1 Hz, 3 x CH), 134.47 (C), 133.57 (d, *J* = 9.9 Hz, 3 x CH), 132.67 (CH), 131.71 (CH), 130.55 (d, *J* = 12.5 Hz, 6 x CH), 128.82 (C), 125.75 (CH), 118.15 (d, *J* = 86.0 Hz, 3 x C), 38.88 (CH_2_), 27.12 (d, *J* = 16.8 Hz, CH_2_), 27.10 (CH_2_), 22.72 (d, *J* = 50.4 Hz, CH_2_), 21.65 (d, *J* = 4.4 Hz, CH_2_). ^31^P NMR (162 MHz, CDCl_3_): δ_P_ 23.98 (1P, s). HRMS (ESI^+^): ${\text{C}}_{29}{\text{H}}_{27}^{35}\text{CI}{\text{N}}_{2}{\text{o}}_{3}\text{p}$ requires 531.1599 found 531.1606 (M^+^).

#### 4.1.9 {5-[(2′-Chloro-5′-nitrophenyl)formamido]pentyl} triphenylphosphonium chloride 7b

1-Ethyl-3-(3-dimethylaminopropyl) carbodiimide hydrochloride (169 mg, 0.844 mmol, 1.50 eq.) was added to a stirring solution of 2-chloro-5-nitrobenzoic acid **10b** (154 mg, 0.766 mmol, 1.30 eq.) and *N*-hydroxysuccinimide (89.4 mg, 0.844 mmol, 1.50 eq.) in dichloromethane (4.0 mL) at 0°C. The reaction mixture was allowed to warm to room temperature and stirred for 2 h. A solution of (5-aminopentyl) triphenylphosphonium bromide hydrobromide **9** (300 mg, 0.589 mmol, 1.00 eq.) and triethylamine (246 μL, 1.77 mmol, 3.00 eq.) in dichloromethane (4.0 mL) was added and the reaction mixture was stirred for 19 h at room temperature. The reaction mixture was filtered, and the filtrate was washed with an aqueous solution of HCl (20 mL, 1 N). The aqueous layer was extracted with dichloromethane (3 × 10 mL) and the organic layers were combined, dried with MgSO_4_, and the solvent concentrated *in vacuo*. The crude material was purified by column chromatography [SiO_2_, dichloromethane: methanol 0–20 %] to afford amide **7b** as yellow foam (149 mg, 44 %). υ_max_ (ATR): 3156 (N–H), 1648 (C=O), 1609 (ArC = ArC), 1555 (NO_2_), 1521 (NO_2_) cm^–1^. ^1^H NMR (400 MHz, CDCl_3_): δ_H_ 8.69 (1H, t, *J* = 5.5 Hz, NH), 8.36 (1H, d, *J* = 2.7 Hz, H-6′), 8.11 (1H, dd, *J* = 8.8, 2.7 Hz, H-4′), 7.84 – 7.75 (9H, m, Ph), 7.72 – 7.65 (6H, m, Ph), 7.50 (1H, d, *J* = 8.8 Hz, H-3′), 3.72 – 3.61 (2H, m, CH_2_-1), 3.47 (2H, q, *J* = 5.8 Hz, CH_2_-5), 1.87 – 1.78 (4H, m, CH_2_-4 and CH_2_-3), 1.76 – 1.64 (2H, m, CH_2_-2). ^13^C NMR (101 MHz, CDCl_3_): δ_C_ 165.53 (C), 146.10 (C), 138.37 (C), 137.80 (C), 135.13 (d, *J* = 3.1 Hz, 3 x CH), 133.62 (d, *J* = 10.0 Hz, 6 x CH), 130.90 (CH), 130.55 (d, *J* = 12.6 Hz, 6 x CH), 124.90 (CH), 124.73 (CH), 118.25 (d, *J* = 85.9 Hz, 3 x C), 38.87 (CH_2_), 27.37 (CH_2_), 27.08 (d, *J* = 16.8 Hz, CH_2_), 22.59 (d, *J* = 50.4 Hz, CH_2_), 21.73 (d, *J* = 4.2 Hz, CH_2_). ^31^P NMR (162 MHz, CDCl_3_): δ_P_ 24.29 (1P, s). HRMS (ESI^+^): ${\text{C}}_{29}{\text{H}}_{27}^{35}{\text{ClN}}_{2}{\text{O}}_{3}\text{P}$ requires 531.1599 found 531.1621 (M^+^).

#### 4.1.10 {5-[(5′-Chloro-2′-nitrophenyl)formamido]pentyl} triphenylphosphonium chloride 7c

*N, N*-Dicyclohexylcarbodiimide (294 mg, 1.43 mmol, 1.25 eq.) was added to a stirring solution of 5-chloro-2-nitrobenzoic acid **10c** (230 mg, 1.14 mmol, 1 eq.) and *N*-hydroxysuccinimide (127 mg, 1.25 mmol, 1.10 eq.) in dichloromethane (8.0 mL) at 0°C. The reaction mixture was allowed to warm to room temperature and stirred for 2 h. A solution of (5-aminopentyl)triphenylphosphonium bromide hydrobromide **9** (549 mg, 1.07 mmol, 1.00 eq.) and triethylamine (318 μL, 2.28 mmol, 2.00 eq.) in anhydrous dichloromethane (2.0 mL) was added and the mixture was stirred at room temperature for 19 h under argon. The mixture was filtered, and the filtrate was washed with an aqueous solution of HCl (20 mL, 1 N). The aqueous layer was extracted with dichloromethane (3 × 10 mL) and the organic layers were combined, dried with MgSO_4_, and the solvent concentrated *in vacuo*. The crude material was purified by column chromatography [SiO_2_, dichloromethane: methanol 0–20 %] to afford amide **7c** as an off-white foam (371 mg, 57 %). υ_max_ (ATR): 1738 (C=O), 1660 (ArC = ArC), 1524 (NO_2_). ^1^H NMR (400 MHz, CDCl_3_): δ_H_ 9.19 (1H, t, *J* = 5.5 Hz, NH), 7.91 (1H, d, *J* = 8.7 Hz, H-3′), 7.84 – 7.73 (9H, m, Ph), 7.73 – 7.63 (7H, m, H-6′ and Ph), 7.41 (1H, dd, *J* = 8.7, 2.3 Hz, H-4′), 3.70 – 3.53 (2H, m, CH_2_-1), 3.47 (2H, q, *J* = 5.8 Hz, CH_2_-5), 1.93 – 1.65 (m, 6H, CH_2_-2, CH_2_-3 and CH_2_-4). ^13^C NMR (101 MHz, CDCl_3_): δ_C_ 165.78 (C), 145.05 (C), 140.11 (C), 135.25 (C), 135.15 (d, *J* = 3.0 Hz, 3 x CH), 133.70 (d, *J* = 9.9 Hz, 6 x CH), 130.61 (d, *J* = 12.5 Hz, 6 x CH), 129.87 (CH), 129.77 (CH), 125.69 (CH), 118.44 (d, *J* = 85.9 Hz, 3 x C), 38.99 (CH_2_), 27.27 (CH_2_), 27.11 (CH_2_), 22.51 (d, *J* = 50.4 Hz, CH_2_), 21.93 (d, *J* = 4.3 Hz, CH_2_). ^31^P NMR (162 MHz, CDCl_3_): δ_P_ 24.22 (1P, s). HRMS (ESI^+^): ${\text{C}}_{30}{\text{H}}_{29}^{35}{\text{ClN}}_{2}{\text{O}}_{3}\text{P}$ requires 531.1599 found 531.1614 (M^+^).

#### 4.1.11 (5-Aminopentyl)triphenylphosphonium bromide hydrobromide 9

A solution of 5-amino-1-pentanol **8** (0.530 g, 5.10 mmol, 1.00 eq) in aqueous hydrogen bromide (48 % w/v in water, 5.00 mL) was stirred for 48 h at reflux. The reaction mixture was allowed to cool to room temperature and the solvent concentrated *in vacuo*. Recrystallisation of the crude material from ethanol and ethyl acetate afforded 5-bromopentylammonium bromide as a brown hygroscopic solid (828 mg, 66 %). υ_max_ (ATR): 2940 (N–H) cm^–1^. ^1^H NMR (400 MHz, CD_3_OD): δ_H_ 3.47 (2H, t, *J* = 6.6 Hz, C, CH_2_-5), 2.98 – 2.89 (2H, m, CH_2_-1), 1.96 – 1.83 (2H, m, CH_2_-4), 1.75 – 1.61 (2H, m, CH_2_-2), 1.61 – 1.48 (2H, m, CH_2_-3). ^13^C NMR (101 MHz, CD_3_OD): δ_C_ 39.19 (CH_2_), 32.38 (CH_2_), 31.86 (CH_2_), 26.33 (CH_2_), 24.62 (CH_2_). HRMS (ESI^+^): ${\text{C}}_{5}{\text{H}}_{13}^{79}$BrN requires 166.0226 found 166.0227 (MH^+^). The ^1^H and ^13^C NMR data was in agreement with that previously reported.^56^ Triphenylphosphine (1.70 g, 6.48 mmol, 2.00 eq.) was added to a solution of 5-bromopentylammonium bromide (800 mg, 3.24 mmol, 1.00 eq) in acetonitrile (20.0 mL) and the reaction mixture was stirred for 48 h at reflux. The reaction mixture was allowed to cool to room temperature and the solvent concentrated *in vacuo*. The crude material was redissolved in chloroform and triturated three times with diethyl ether to afford (5-aminopentyl)triphenylphosphonium bromide hydrobromide **9** as a white hygroscopic solid (2.89 g, 92 %). υ_max_ (ATR): 2909 (N–H) cm^−1^. ^1^H NMR (400 MHz, CDCl_3_): δ_H_ 8.19 (3H, br s, NH_2_), 7.90 – 7.63 (15H, m, Ph), 3.80 – 3.64 (2H, m, CH_2_-1), 3.18 – 2.94 (2H, m, CH_2_-5), 2.08 – 1.92 (2H, m, CH_2_-2), 1.92 – 1.76 (2H, m, CH_2_-4), 1.76 – 1.59 (2H, m, CH_2_-3). ^13^C NMR (101 MHz, CDCl_3_): δ_C_ 135.16 (d, *J* = 2.9 Hz, 3 x CH), 133.78 (d, *J* = 10.0 Hz, 6 x CH), 130.63 (d, *J* = 12.5 Hz, 6 x CH), 118.15 (d, *J* = 86.1 Hz, C x 3), 39.38 (CH_2_), 26.82 (d, *J* = 16.5 Hz, CH_2_), 25.66 (CH_2_), 22.32 (d, *J* = 50.9 Hz (CH_2_), 21.63 (CH_2_). ^31^P NMR (162 MHz, CDCl_3_): δ_P_ 24.25. HRMS (ESI^+^): C_23_H_28_NP requires 174.5974 found 174.5981 (M^2+^). The ^1^H and ^13^C NMR data was in broad agreement with that previously reported in CDCl_3_.^57^

#### 4.1.12 Methyl 4-(S-Glutathionyl)-3-nitrobenzoate 11a

The reaction was performed as described in the general procedure using chloronitrobenzene derivative **6a** (100 mg, 0.464 mmol). The reaction mixture was stirred at room temperature for 24 h. Purification by RP-flash column chromatography [water:acetonitrile 0–100 %] afforded the glutathione adduct **11a** as a yellow solid (195 mg, 86 %). υ_max_ (ATR): 1723 (C=O). 1603 (ArC = ArC), 1516 (NO_2_) cm^–1. 1^H NMR (400 MHz, D_2_O): δ_H_ 8.69 (1H, d, *J* = 1.6 Hz, H-2), 8.17 (1H, dd, *J* = 8.5, 1.6 Hz, H-6), 7.74 (1H, d, *J* = 8.6 Hz, H-5), 4.75 (1H, dd, *J* = 9.3, 4.8 Hz, Cys CH), 3.99 – 3.95 (3H, s, OCH_3_), 3.82 – 3.66 (4H, m, Gly CH_2_, Gln CH, and CH_A_C*H*_*B*_S), 3.40 (1H, dd, *J* = 14.2, 9.3 Hz, C*H*_*A*_CH_B_S), 2.47 (2H, t, *J* = 6.6 Hz, C*H*_*2*_CONH), 2.08 (2H, q, *J* = 7.2 Hz, C*H*_*2*_CH_2_CONH). ^13^C NMR (101 MHz, D_2_O): δ_C_ 176.13 (C), 174.90 (C), 174.36 (C), 171.03 (C), 166.77 (C), 145.63 (C), 141.43 (C), 133.81 (CH), 127.55 (CH), 127.13 (CH), 126.86 (C), 54.16 (CH), 53.01 (CH_3_), 51.71 (CH), 43.35 (CH_2_), 33.34 (CH_2_), 31.42 (CH_2_), 26.42 (CH_2_). HRMS (ESI^+^): C_18_H_23_N_4_O_10_S requires 487.1129 found 487.1137 (MH^+^). Assignment of ^1^H and ^13^C NMR signals was supported by analysis of COSY, HSQC and DEPT experiments.

#### 4.1.13 Methyl 2-(S-Glutathionyl)-4-nitrobenzoate 11b

The reaction was performed as described in the general procedure using chloronitrobenzene derivative **6b** (100 mg, 0.464 mmol). The reaction mixture was stirred at room temperature for 24 h. Purification by RP-flash column chromatography [water:acetonitrile 0–100 %] afforded the glutathione adduct **11b** as a pale-orange solid (105 mg, 47 %). υ_max_ (ATR): 1718 (C=O), 1645 (C=O), 1598 (ArC = ArC), 1507 (NO_2_) cm^−1^. ^1^H NMR (400 MHz, D_2_O): δ_H_ 8.71 (1H, d, *J* = 2.6 Hz, H-3), 8.33 (1H, dd, *J* = 9.1, 2.7, H-5), 7.69 (1H, d, *J* = 9.0 Hz, H-6), 4.76 (1H, dd, *J* = 9.2, 4.7 Hz, Cys CH), 3.96 (3H, s, *J* = 1.0 Hz, OCH_3_), 3.83 – 3.63 (4H, m, Gly CH_2_, Gln CH and SCH_A_C*H*_*B*_), 3.38 (1H, dd, *J* = 13.9, 9.1 Hz, SC*H*_*A*_CH_B_), 2.47 (2H, t, *J* = 7.8 Hz, C*H*_*2*_CONH), 2.14 – 2.02 (2H, m, C*H*_*2*_CH_2_CONH). ^13^C NMR (101 MHz, D_2_O): δ_C_ 176.13 (C), 174.90 (C), 174.39 (C), 171.12 (C), 166.61 (C), 148.78 (C), 144.08 (C), 127.53 (C), 126.88 (CH), 126.61 (CH), 126.45 (CH), 54.18 (CH), 53.08 (CH_3_), 51.83 (CH), 43.37 (CH_2_), 33.04 (CH_2_), 31.47 (CH_2_), 26.45 (CH_2_). HRMS (ESI^+^): C_18_H_23_N_4_O_10_S requires 487.1129 found 487.1139 (MH^+^). Assignment of ^1^H and ^13^C NMR signals was supported by analysis of 2-dimensional COSY, HSQC and DEPT experiments.

#### 4.1.14 Methyl 5-(S-Glutathionyl)-2-nitrobenzoate 11c

The reaction was performed as described in the general procedure using chloronitrobenzene derivative **6c** (100 mg, 0.464 mmol). The reaction mixture was stirred at room temperature for 24 h. Purification by RP-flash column chromatography [water:acetonitrile 0–100 %] afforded the glutathione adduct **11c** as an off-white solid (195 mg, 86 %). υ_max_ (ATR): 1731 (C=O), 1589 (ArC = ArC), 1522 (NO_2_), 1510 (NO_2_) cm^–1. 1^H NMR (400 MHz, D_2_O): δ_H_ 8.12 – 8.05 (1H, m, CH-3), 7.74 – 7.67 (2H, m, H-4 and H-6), 4.71 (1H, dd, *J* = 8.0, 5.2 Hz, Cys CH), 3.98 (3H, s, OCH_3_), 3.76 – 3.60 (4H, m, Gly CH_2_, Gln CH, and CH_A_C*H*_*B*_S), 3.46 (1H, dd, *J* = 14.6, 8.2 Hz, C*H*_*A*_CH_B_S), 2.49 – 2.36 (2H, m, C*H*_*2*_CONH), 2.06 (2H, q, *J* = 7.3 Hz, C*H*_*2*_CH_2_CONH). ^13^C NMR (101 MHz, D_2_O): δ_C_ 176.05 (C), 174.86 (C), 174.59 (C), 170.99 (C), 168.22 (C), 144.99 (C), 143.69 (C), 130.27 (CH), 128.11 (C), 127.44 (CH), 125.17 (CH), 54.21 (CH), 54.03 (CH_3_), 52.58 (CH), 43.29 (CH_2_), 33.33 (CH_2_), 31.45 (CH_2_), 26.48 (CH_2_). HRMS (ESI^+^): C_18_H_23_N_4_O_10_S requires 487.1129 found 487.1136 (MH^+^). Assignment of ^1^H and ^13^C NMR signals was supported by analysis of COSY, HSQC and DEPT experiments.

#### 4.1.15 {5-[(4′-{S-Glutathionyl}-3′-nitrophenyl)formamido]pent-1-yl} triphenylphosphonium chloride 12a

The reaction was performed as described in the general procedure using chloronitrobenzene derivative **7a** (58 mg, 0.10 mmol). The reaction mixture was stirred at room temperature for 3 h. Purification by RP-flash column chromatography [water:acetonitrile 0–100 %] afforded the glutathione adduct **12a** as a yellow foam (47 mg, 55 %). υ_max_ (ATR): 2970 (CH), 1739 (C=O), 1637 (ArC = ArC), 1604 (ArC = ArC), 1516 (NO_2_) cm^–1^. ^1^H NMR (400 MHz, CD_3_OD): δ_H_ 8.54 (1H, d, *J* = 2.0 Hz, H-2′), 8.09 (1H, dd, *J* = 8.5, 2.0 Hz, H-6′), 7.91 – 7.70 (16H, m, Ph and H-5′), 4.70 (1H, dd, *J* = 8.4, 5.2 Hz, Cys CH), 3.76 (2H, s, Gly CH_2_), 3.66 (1H, dd, *J* = 14.0, 5.2 Hz, CH_A_C*H*_*B*_S), 3.60 (1H, t, *J* = 6.1 Hz, Gln CH), 3.50 – 3.25 (5H, m, C*H*_*A*_CH_B_S, CH_2_-1 and CH_2_-5), 2.59 – 2.40 (2H, m, C*H*_*2*_CONH), 2.10 (2H, q, *J* = 7.0 Hz, C*H*_*2*_CH_2_CONH), 1.81 – 1.60 (6H, m, CH_2_-2, CH_2_-3 and CH_2_-4). ^13^C NMR (101 MHz, CD_3_OD): δ_C_ 176.10 (C), 175.35 (C), 174.53 (C), 171.22 (C), 166.81 (C), 147.01 (C), 141.65 (C), 136.26 (d, *J* = 3.0 Hz, 3 x CH), 134.83 (d, *J* = 10.0 Hz, 6 x CH), 132.82 (CH), 132.27 (C), 131.52 (d, *J* = 12.6 Hz, 6 x CH), 128.40 (CH), 126.06 (CH), 119.99 (d, *J* = 86.3 Hz, 3 x C), 55.63 (CH), 53.57 (CH), 44.72 (CH_2_), 40.50 (CH_2_), 34.58 (CH_2_), 33.07 (CH_2_), 29.54 (CH_2_), 28.96 (d, *J* = 16.8 Hz, CH_2_), 28.15 (CH_2_), 23.31 (d, *J* = 3.9 Hz, CH_2_), 22.72 (d, *J* = 51.1 Hz, CH_2_). ^31^P NMR (162 MHz, CD_3_OD): δ_P_ 23.71 (1P, s). HRMS (ESI^+^): C_40_H_45_N_5_O_9_PS requires 802.2670 found 802.2676 (M^+^). Assignment of ^1^H and ^13^C NMR signals was supported by analysis of COSY, HSQC and DEPT experiments.

#### 4.1.16 {5-[(2′-{S-Glutathionyl}-5′-nitrophenyl)formamido]pent-1-yl} triphenylphosphonium chloride 12b

The reaction was performed as described in the general procedure using chloronitrobenzene derivative **7b** (40 mg, 0.071 mmol). The reaction mixture was stirred at room temperature for 3 h. Purification by RP-flash column chromatography [water:acetonitrile 0–100 %] afforded the glutathione adduct **12b** as an orange foam (27.8 mg, 47 %). υ_max_ (ATR): 3379 (N–H), 1646 (C=O), 1595 (ArC = ArC), 1507 (NO_2_) cm^–1. 1^H NMR (400 MHz, D_2_O): δ_H_ 8.23 (1H, dd, *J* = 8.8, 2.5 Hz, H-4′), 8.06 (1H, d, *J* = 2.5 Hz, H-6′), 7.86 – 7.59 (16H, m, Ph and H-3′), 4.59 (1H, dd, *J* = 8.5, 5.2 Hz, Cys CH), 3.73 – 3.53 (3H, m, Gly CH_2_ and SCH_A_C*H*_*B*_), 3.42 – 3.18 (6H, m, Gln CH, SCH_A_C*H*_*B*_, CH_2_-1 and CH_2_-5), 2.38 – 2.20 (2H, m, C*H*_*2*_CONH), 1.92 – 1.79 (2H, m, C*H*_*2*_CH_2_CONH), 1.78 – 1.67 (2H, m, CH_2_-2), 1.66 – 1.50 (4H, m, CH_2_-3 and CH_2_-4). ^13^C NMR (101 MHz, D_2_O): δ_C_ 175.88 (C), 175.39 (C), 170.78 (C), 168.40 (C), 144.56 (C), 143.98 (C), 135.43 (C), 134.96 (d, *J* = 2.6 Hz, 3 x CH), 133.44 (d, *J* = 10.0 Hz, 6 x CH), 130.03 (d, *J* = 12.5 Hz, 6 x CH), 127.77 (CH), 125.30 (CH), 122.48 (CH), 118.07 (d, *J* = 86.6 Hz, 3 x C), 54.91 (CH), 52.29 (CH), 43.32 (CH_2_), 39.11 (CH_2_), 33.12 (CH_2_), 31.95 (CH_2_), 28.82 (CH_2_), 27.40 (CH_2_), 27.22 (d, *J* = 16.0 Hz, CH_2_), 21.72 (d, *J* = 51.9 Hz, CH_2_), 21.37 (d, *J* = 4.1 Hz, CH_2_). ^31^P NMR (162 MHz, D_2_O): δ_P_ 23.80 (1P, s). HRMS (ESI^+^): C_40_H_45_N_5_O_9_PS requires 802.2670 found 802.2684 (M^+^). Assignment of ^1^H and ^13^C NMR signals was supported by analysis of COSY, HSQC and DEPT experiments. One signal corresponding to a C was not identified in the ^13^C NMR spectrum, likely due to coincidence with another signal.

#### 4.1.17 {5-[(5′-{S-Glutathionyl}-2′-nitrophenyl)formamido]pent-1-yl} triphenylphosphonium chloride 12c

The reaction was performed as described in the general procedure using chloronitrobenzene derivative **7c** (70.0 mg, 0.123 mmol). The reaction mixture was stirred at room temperature for 3 h. Purification by RP-flash column chromatography [water:acetonitrile 0–100 %] afforded the glutathione adduct **12c** as a yellow foam (40.6 mg, 47 %). υ_max_ (ATR): 1739 (C=O), 1659 (ArC = ArC), 1521 (NO_2_) cm^−1^. ^1^H NMR (400 MHz, CD_3_OD): δ_H_ 8.02 1H, d, *J* = 8.6 Hz, H-3′), 7.93 – 7.71 (15H, m, Ph), 7.60 (1H, dd, *J* = 8.8, 2.1 Hz, H-4′), 7.47 (1H, d, *J* = 2.1 Hz, H-6′), 4.64 (1H, dd, *J* = 8.6, 5.0 Hz, Cys CH), 3.74 – 3.57 (4H, m, Gly CH_2_, Gln CH and CH_A_C*H*_*B*_S), 3.51 – 3.39 (2H, m, CH_2_-1), 3.39 – 3.32 (3H, m, CH_2_-5 and C*H*_*A*_CH_B_S), 2.57 – 2.40 (2H, m, C*H*_*2*_CONH), 2.14 – 2.03 (2H, m, C*H*_*2*_CH_2_CONH), 1.82 – 1.60 (6H, m, CH_2_-2, CH_2_-3 and CH_2_-4). ^13^C NMR (101 MHz, CD_3_OD): δ_C_ 175.80 (C), 175.36 (C), 171.27 (C), 168.94 (C), 146.81 (C), 144.95 (C), 136.27 (d, *J* = 3.1 Hz, 3 x CH), 134.85 (d, *J* = 10.0 Hz, 6 x CH), 134.76 (C), 131.55 (d, *J* = 12.6 Hz, 6 x CH), 129.54 (CH), 127.70 (CH), 126.21 (CH), 120.00 (d, *J* = 86.4 Hz, 3 x C), 55.53 (CH), 53.86 (CH), 44.56 (CH_2_), 40.08 (CH_2_), 34.53 (CH_2_), 33.18 (CH_2_), 29.12 (CH_2_), 28.58 (d, *J* = 17.0 Hz, CH_2_), 27.89 (CH_2_), 23.11 (d, *J* = 4.0 Hz, CH_2_), 22.74 (d, *J* = 51.6 Hz, CH_2_). ^31^P NMR (162 MHz, DMSO): δ_P_ 23.99 (1P, s). HRMS (ESI^+^): C_40_H_45_N_5_O_9_PS requires 802.2670 found 802.2676 (M^+^). Assignment of ^1^H and ^13^C NMR signals was supported by analysis of COSY, HSQC and DEPT experiments. One signal corresponding to a C was not identified in the ^13^C NMR spectrum, likely due to coincidence with another signal.

#### 4.1.18 Cloning, expression and purification of human GST proteins DNA Sequences

The hGST A-1, A4 and K1 DNA sequences were purchased as GeneArt Strings double stranded DNA fragments from ThermoFisher Scientific. The genes were codon optimised for expression in *Escherichia coli* and included 5′ and 3′, 15 nucleotide long sequences complementary to the cloning vector. The genes were cloned into the expression vector pNIC-28-*Bsa*I by ligation independent cloning (LIC).^58^.

The genes were treated with T4 DNA polymerase in the presence of dCTP to generate sticky ends. The pNIC-28-*Bsa*I vector was linearized using the restriction enzyme *Bsa*I and treated with T4 DNA polymerase in the presence of dGTP to generate sticky ends complementary to those on the synthesised genes. The individual T4 DNA polymerase treated genes and vector were added together and annealed by incubation at 18 °C for 10 min. and transformation into into *E. coli* DH5a competent cells and grown on Luria-Bertani (Lennox) broth^53^ agar plates containing 30 µg/mL kanamycin overnight as the selection antibiotic. Single colonies were subsequently grown in liquid Luria-Bertani broth^53^ with Kanamycin. The plasmid DNA from the pelleted bacteria was extracted using an alkaline lysis and spin-column based kit and sent for Sanger sequencing to confirm successful cloning.

The recombinant DNA were transformed into *E.coli* BL21 (DE3) competent cells, single colonies grown on LB agar plates with 30 µg/mL Kanamycin were picked and grown in 10 mls LB liquid media overnight. 5 mLs of culture were added to 300 mls of Terrific Broth (TB) medium with 30 µg/mL Kanamycin and grown at 37 °C with shaking at 220 rpm until cell density reached an OD_600_ of approximately 0.8. The cultures were cooled on ice for 5 min and protein expression was induced with 0.1 mM of isopropy1-β-d-thiogalactopyranoside (IPTG), and the cultures grown overnight at 15 °C. The bacteria were harvested by centrifugation at 4000 rpm (3400g) for 20 min with a Sigma 4 K15 centrifuge at 20 °C and the cell pellet resuspended in lysis buffer (50 mM Tris-HCl, 150 mM NaCl buffer, pH 8.0). The bacteria were lysed by sonification (20 kHz, 125 W at half power, 30 s on with 15 s off for 30 cycles) on ice, the lysate was then centrifuged at 24,000 rpm, 50870g with a Sigma 3 K30 centrifuge at 4 °C for 30 min to separate the soluble fraction was separated from the insoluble cell material. The soluble fraction was loaded onto a gravity flow Ni-NTA agarose column and the proteins purified by immobilized nickel ion affinity chromatography. The column was first equilibrated with 20 mL of Buffer A (50 mM Tris-HCl, 250 mM NaCl, pH 8.0). The protein solution was then passed through the column and the flow through collected. The column was washed with a further 5 mL of Buffer A and collected with the flow through. The column was then washed with 10 mLs of Buffers B (50 mM Tris-HCl, 250 mM NaCl, 50 mM Imidazole, pH 8.0), C (50 mM Tris-HCl, 250 mM NaCl, 75 mM Imidazole, pH 8.0) and D (50 mM Tris-HCl, 250 mM NaCl, 250 mM Imidazole, pH 8.0) and these fractions collected in separate containers. The concentration and activity of fractions from Buffer B, C and D were analysed using a nanodrop ND-1000 spectrophotometer and the purity was evaluated by SDS PAGE. Fractions eluted with Buffer D contained the most pure protein and was dialysed against storage buffer (50 mM Tris HCl, at pH 8.0) and concentration was measured by UV absorption at 280 nm (using ε280nm = 1.291, 0.772 and 0.665 for GSTs K1-1, A1-1 and A4-4, respectively) before storage at –20 °C in 50 % glycerol with dithiothreitol (DTT) added as a reducing agent. Each GST enzyme was obtained in high yield with 28 mg of GSTK1-1, 59 mg of GSTA1-1, and 42 mg of GSTA4-4, recovered from 0.6 L cultures. The vector derived 6xHistidine-tag and TEV (Tobacco Etch Virus Protease) recognition sequence were not cleaved from the proteins.

### 4.2 UV/Vis reaction rate measurements and calculation of Michaelis constants

UV–Vis measurements and kinetic assays were carried out using a Jasco V550 UV/Vis spectrometer at 30 °C using quartz cuvettes ranging from 0.3 to 1 mL in volume and 0.1–1 cm in pathlength. All assays were completed using either a 0.1 M sodium phosphate buffer, pH 6.5 (diluted from a 1 M stock mixing 1 M NaH_2_PO_4_ and 1 M Na_2_HPO_4_ in a proportion of 68.5 to 31.5), or a 0.1 M HEPES-HCl buffer, pH 8. The reaction rate was monitored by measuring the initial rate of formation of the glutathione conjugate.

To fully characterise the enzymes with respect to both CDNB and GSH, the concentration of one substrate was kept constant at near saturating concentrations, while the concentration of the other substrate was varied. When studying CDNB and the other chloronitrobenzene derivatives, GSH concentration was fixed at 10 mM to ensure saturation. However, when studying GSH, CDNB concentration was fixed at 1 mM owing to its poor aqueous solubility above this concentration, which for some assays, was likely insufficient to saturate the enzyme. Hence, for assays where saturating conditions were unlikely achieved, the associated kinetic constants are described as ‘apparent’.

To calculate the background chemical rates and kinetic constants for the various substrates a suitable absorption wavelength and molar extinction coefficient needed to be obtained for each substrate. The UV absorption spectra were measured between 220 nm and 600 nm for a given substrate and its GS-conjugate, both at 100 µM. From the spectra a suitable wavelength for measurements with minimal overlap between the substrate and GS-conjugate spectra was identified. The molar extinction coefficients for the chloronitrobenzene derivatives were as follows: CDNB **1**, 0.578 mM^–1^ cm^–1^; MitoCDNB **4**, 7.88 mM^–1^ cm^–1^ at 328 nm; **6a**, 2.05 mM^–1^ cm^–1^ at 286 nm; **6b**, 0.704 mM^–1^ cm^–1^ at 343 nm; **6c**, 0.780 mM^–1^ cm^–1^ at 348 nm; **7a**, 1.16 mM^–1^ cm^–1^ at 284 nm; **7b**, 0.687 mM^–1^ cm^–1^ at 342 nm; **7c**, 0.979 mM^–1^ cm^–1^ at 345 nm. The molar extinction coefficients for the GS-conjugates were as follows: GSDNB **3**, 9.60 mM^–1^ cm^–1^ at 340 nm; MitoGSDNB **5**, 17.3 mM^–1^ cm^–1^ at 328 nm; **11a**, 11.4 mM^–1^ cm^–1^ at 286 nm; **11b**, 14.1 mM^–1^ cm^–1^ at 343 nm; **11c**, 7.48 mM^–1^ cm^–1^ at 348 nm; **12a**, 7.88 mM^–1^ cm^–1^ at 284 nm; **12b**, 11.2 mM^–1^ cm^–1^ at 342 nm; **12c**, 5.91 mM^–1^ cm^–1^ at 345 nm. Ideally, the absorbance of the substrate was also low at this wavelength. This wavelength was selected to measure the absorbance kinetics for the formation of the GS-conjugate from the substrate. All assay mixtures were prepared and mixed by shaking *in situ* and the rate of the reaction was measured for at least 1 min. The initial rates of reaction were determined using the Jasco V550 software choosing initial data to maximise the correlation with a straight line. Background chemical rates were measured once at pH 6.5, while at pH 8.0, the chemical rate was appreciable and were measured in triplicate. Enzyme catalysed reaction rates were measured at a variety of substrate concentrations in triplicate. For a given concentration of the substrate of interest, the averaged background rate was subtracted from the averaged enzyme-catalysed reaction rate to obtain the real enzyme-catalysed reaction rate in A s^–1^. Using the Beer-Lambert Law and the difference in molar extinction coefficients between the substrate and corresponding GS-conjugate, the real enzyme-catalysed rate was converted to mM s^−1^. The values of *K*_*M*_ and *k*_*cat*_ were obtained by fitting the initial rate data to the Michaelis-Menten equation using non-linear regression in Microsoft Excel for compounds where non-linear behaviour was observed,^59^ or to the Lineweaver-Burk model where linear (or near-linear) behaviour was observed. For constants determined by non-linear regression, standard errors were calculated using the method of *Kitaoka*.^60^ For constants determined by linear regression, standard errors were calculated using the LINEST function in Microsoft Excel.

## Appendix A. Supplementary data

A figure showing the SDS PAGE gel of the purified hGSTs, UV-Vis Absorbance spectra, kinetics graphs for GSTs with respect to every substrate, and the processed NMR spectra are available as supplementary information. Supplementary data to this article can be found online at https://doi.org/10.1016/j.bmc.2024.117712.

## Figures and Tables

**Table 1:** Kinetic constants of human GST enzymes for CDNB and GSH at pH 6.5 and 8.0, at 30°C. When measuring kinetics for CDNB, for GSTs A1-1, A4-4, and K1-1, the saturating substrate, GSH was fixed at 10 mM, respectively. When measuring kinetics for GSH, the saturating substrate, CDNB was fixed at 1 mM. Background reaction initial rates were subtracted from the enzyme-catalysed reaction initial rates prior to calculation of the kinetic constants. Kinetic constants were calculated based on molecular weights of 28184.80, 28256.83 and 28049.58 Da and stock concentrations of 2.91, 2.11 and 1.42 mg mL^−1^ for GSTs A1-1, A4-4 and K1-1, respectively. *Apparent kinetic constants were calculated for GSH because the enzyme could not be saturated with CDNB 1 due to its low aqueous solubility: ${\text{k}}_{\text{cat}}^{\text{app}},{\text{K}}_{\text{M}}^{\text{app}}$ and (k_cat_ /K_M_)^app^. ^#^1 mM^−1^s^−1^ = 10^3^ M^−1^s^−1^. NM = Not measured.

		hGSTK1-1			hGSTA1-1			hGSTA4-4		
Substrate	PH	*k_cat_* (s^–^^1^)	*K_M_* (mM)	*k_cat_* / *K_M_* (mM^–^^1^s^–^^1^)^#^	*k_cat_* (s^–^^1^)	*K_M_* (mM)	*k_cat_* / *K_M_* (mM^–^^1^s^–^^1^)^#^	*k_cat_* (s^–^^1^)	*K_M_* (mM)	*k_cat_* / *K_M_* (mM^–^^1^s^–^^1^)^#^
**CDNB**	6.5	NM	NM	NM	35.4 ± 0.9	0.492 ± 0.036	71.9 ± 3.7	14.9 ± 2.4	5.20 ± 1.1	2.93 ± 0.15
	8.0	14.6 ± 1.5	1.90 ± 0.32	7.67 ± 0.53	45.3 ± 1.7	0.128 ± 0.018	353 ± 38	4.94 ± 0.36	0.253 ± 0.052	19.5 ± 2.7
GSH*	6.5	NM	NM	NM	36.1 ± 1.0	0.276 ± 0.034	130 ± 13	1.80 ± 0.05	1.14 ± 0.99	1.59 ± 0.11
	8.0	4.83 ± 0.07	0.652 ± 0.048	7.41 ± 0.48	49.1 ± 0.9	0.233 ± 0.024	211 ± 19	6.10 ± 0.23	0.580 ± 0.85	10.5 ± 1.2

**Table 2:** Second-order background rate constants k’ of the compounds for the reaction with GSH at 10 mM at pH 8 at 30°C. For a given substrate, a mean rate constant k’ was calculated from a series of four rate constants determined at four different concentrations. Data are expressed as means ± S.D. (n = 4) of four rate constants. ^#^1 mM^−1^s^−1^ = 10^3^ M^−1^s^−1^. ND = Not detected.

Compound	Substituents				Background rate constant *k’* (mM^–^^1^s^–^^1^)^#^
	2-	3-	4-	5-	
**CDNB 1**	NO_2_	H	NO_2_	H	(5.05 ± 0.40) x 10^–^^5^
**MitoCDNB 4**	NO_2_	H	NO_2_	NH(CH_2_)_4_TPP	(1.50 ± 0.18) x 10^–^^5^
**6a**	NO_2_	H	CO_2_CH_3_	H	(2.92 ± 0.10) x 10^–^^6^
**7a**	NO_2_	H	CONH(CH_2_)_5_TPP	H	ND
**6b**	CO_2_CH_3_	H	NO_2_	H	ND
**7b**	CONH(CH_2_)_5_TPP	H	NO_2_	H	ND
**6c**	H	CO_2_CH_3_	NO_2_	H	ND
**7c**	H	CONH(CH_2_)_5_TPP	NO_2_	H	ND

**Table 3:** Catalytic efficiency k_cat_/K_M_ calculated for the compounds for the GST-catalysed reaction with GSH at pH 8 and 30°C. GSH concentration was fixed at 10 mM. Where necessary, enzyme concentrations were varied to observe a reaction with a measurable initial rate and accounted for in any calculations. All measurements were done in triplicate and errors in *k*_*cat*_/*K*_*M*_ reported as ± 1 standard deviation. Kinetic parameters were calculated either by (a) non-linear curve fit to the Michaelis–Menten plot or (b) best line fit to the double reciprocal Lineweaver-Burk plot. All plots provided in supplementary information. ^#^1 mM^−1^s^−1^ = 10^3^ M^−1^s^−1^. ND = Not detected.

Compound	Substituents				*k_cat_/K_M_* (mM^–^^1^s^–^^1^)^#^		
	2-	3-	4-	5-	hGSTA1-1	hGSTA4-4	hGSTK1-1
**CDNB**	NO_2_	H	NO_2_	H	353 ± 38^(a)^	19.5 ± 2.7 ^(a)^	7.67 ± 0.53 ^(a)^
**MitoCDNB**	NO_2_	H	NO_2_	NH(CH_2_)_4_TPP	101 ± 10^(a)^	32.3 ± 1.5^(a)^	0.287 ± 0.038^(a)^
**6a**	NO_2_	H	CO_2_CH_3_	H	4.32 ± 0.28 ^(b)^	0.146 ± 0.003 ^(b)^	0.351 ± 0.010^(b)^
**7a**	NO_2_	H	CONH(CH_2_)_5_TPP	H	21.6 ± 0.5^(b)^	2.57 ± 0.02^(b)^	ND^(c)^
**6b**	CO_2_CH_3_	H	NO_2_	H	1.38 ± 0.04^(b)^	(5.91 ± 0.17) x 10^–2 (b)^	(6.13 ± 0.12) x 10^–2 (b)^
**7b**	CONH(CH_2_)_5_TPP	H	NO_2_	H	(6.49 ± 0.16) x 10^–2(b)^	ND	ND
**6c**	H	CO_2_CH_3_	NO_2_	H	ND	ND	ND
**7c**	H	CONH(CH_2_)_5_TPP	NO_2_	H	ND	ND	ND

**Table 4:** Catalytic rate constant *k*_*cat*_ calculated for the compounds for the GST-catalysed reaction with GSH at pH 8 and 30°C. GSH concentration was fixed at 10 mM. Where necessary, enzyme concentrations were varied to observe a reaction with a measurable initial rate and accounted for in any calculations. All measurements were done in triplicate and errors in *k*_*ca*t_/*K*_*M*_ reported as ± 1 standard deviation. Kinetic parameters were calculated either by (a) non-linear curve fit to the Michaelis–Menten plot or (b) best line fit to the double reciprocal Lineweaver-Burk plot. All plots provided in supplementary information. ND = Not detected.

Compound	Substituents				*k_cat_* (S^–1^)		
	2-	3-	4-	5-	hGSTA1-1	hGSTA4-4	hGSTK1-1
**CDNB**	NO_2_	H	NO_2_	H	45.3 ± 1.7 ^(a)^	4.94 ± 0.36 ^(a)^	14.6 ± 1.5 ^(a)^
**MitoCDNB**	NO_2_	H	NO_2_	NH(CH_2_)_4_TPP	14.7 ± 0.6^(a)^	4.59 ± 0.08^(a)^	0.103 ± 0.008^(a)^
**6a**	NO_2_	H	CO_2_CH_3_	H	8.85 ± 2.79^(b)^	0.521 ± 0.203^(b)^	0.106 ± 0.008^(b)^
**7a**	NO_2_	H	CONH(CH_2_)_5_TPP	H	9.50 ± 0.45^(b)^	2.13 ± 0.05^(b)^	ND
**6b**	CO_2_CH_3_	H	NO_2_	H	2.31 ± 0.55^(b)^	(5.61 ± 0.37) x 10^–2 (b)^	(9.45 ± 0.68) x 10^–2 (b)^
**7b**	CONH(CH_2_)_5_TPP	H	NO_2_	H	(4.67 ± 0.19) x 10^–2 (b)^	ND	ND
**6c**	H	CO_2_CH_3_	NO_2_	H	ND	ND	ND
**7c**	H	CONH(CH_2_)_5_TPP	NO_2_	H	ND	ND	ND

**Table 5:** Michaelis-Menten constant *K*_*M*_ calculated for the compounds for the GST-catalysed reaction with GSH at pH 8 and 30°C. Substrate concentration was varied between 0.0625 and 2 mM, while GSH concentration was fixed at 10 mM. Where necessary, enzyme concentrations were varied to observe a reaction with a measurable initial rate and accounted for in any calculations. All measurements were done in triplicate and errors in *k*_*ca*t_/*K*_*M*_ reported as ± 1 standard deviation. Kinetic parameters were calculated either by (a) non-linear curve fit to the Michaelis–Menten plot or (b) best line fit to the double reciprocal Lineweaver-Burk plot. All plots provided in supplementary information. ND = Not detected.

Compound	Substituents				*K_M_* (mM)		
	2-	3-	4-	5-	hGSTA1-1	hGSTA4-4	hGSTK1-1
**CDNB**	NO_2_	H	NO_2_	H	0.128 ± 0.018 ^(a)^	0.253 ± 0.052 ^(a)^	1.90 ± 0.32 ^(a)^
**MitoCDNB**	NO_2_	H	NO_2_	NH(CH_2_)_4_TPP	0.146 ± 0.020^(a)^	0.142 ± 0.009^(a)^	0.360 ± 0.067^(a)^
**6a**	NO_2_	H	CO_2_CH_3_	H	2.05 ± 0.77^(b)^	3.58 ± 0.09^(b)^	0.301 ± 0.008^(b)^
**7a**	NO_2_	H	CONH(CH_2_)_5_TPP	H	0.441 ± 0.010^(b)^	0.826 ± 0.005^(b)^	ND
**6b**	CO_2_CH_3_	H	NO_2_	H	1.67 ± 0.05^(b)^	0.949 ± 0.027^(b)^	1.54 ± 0.03^(b)^
**7b**	CONH(CH_2_)_5_TPP	H	NO_2_	H	0.720 ± 0.017^(b)^	ND	ND
**6c**	H	CO_2_CH_3_	NO_2_	H	ND	ND	ND
**7c**	H	CONH(CH_2_)_5_TPP	NO_2_	H	ND	ND	ND

## Data Availability

The raw NMR data files for synthesised compounds, the raw data for all absorbance versus time measurements, and the ExCel spreadsheets used to calculate the kinetic parameters in the tables can be found at https://doi.org/10.5525/gla.researchdata.1566.
